# Risk of Dementia After Electroconvulsive Therapy: A Cohort Study on the Population of Wales

**DOI:** 10.1111/acps.70005

**Published:** 2025-06-19

**Authors:** George Kirov, Emily Simmonds, Tyler Kaster, Valentina Escott‐Price

**Affiliations:** ^1^ Division of Psychological Medicine and Clinical Neurosciences, School of Medicine Cardiff University Cardiff UK; ^2^ UK Dementia Research Institute at Cardiff Cardiff University Cardiff UK; ^3^ Temerty Centre for Therapeutic Brain Intervention, Centre for Addiction and Mental Health University of Toronto Toronto Canada; ^4^ Department of Psychiatry University of Toronto Toronto Canada

**Keywords:** affective disorders, dementia, depression, ECT, electroconvulsive, mortality, psychiatric disorder

## Abstract

**Background:**

Electroconvulsive therapy (ECT) is the most effective therapy for severe or treatment‐resistant depression. A common short‐term side effect is memory problems, and it is important to know whether ECT increases the risk for dementia later in life. Major psychiatric disorders are associated with an increased risk for developing dementia, making the analysis of dementia risk challenging. A small number of previous studies indicate that ECT does not increase this risk. We wanted to examine the association between ECT and subsequent risk of dementia in the population of Wales, UK.

**Methods:**

We analysed the electronic health records of the Welsh population. We selected 110,774 people aged between 35 and 65 on 1.1.1995 who had no prior diagnosis of dementia and had been hospitalised with diagnoses of affective disorders. Of those, 1010 received at least one course of ECT between 1995 and 2024 before a diagnosis of dementia.

**Results:**

The 110,774 persons were followed up until the end of the study period in 2024, or the date of dementia diagnosis, or the date of death, for a mean of 24.5 (SD = 6.3) years. 15.4% of the ECT group developed dementia, compared to 13.1% for the non‐ECT‐treated individuals. After controlling for age, sex, social deprivation status, physical comorbidities, history of alcohol abuse, the number of psychiatric hospitalisations and the age when they first occurred, the hazard ratio for dementia was not increased in the ECT group: HR = 0.888, 95% CI: 0.757–1.044, *p* = 0.15.

**Conclusions:**

Though crude analyses found a greater risk of dementia among those receiving ECT, once confounders were accounted for, we failed to find a statistically significant risk for dementia among those who received ECT. Our findings strengthen the conclusions of previous reports and provide further reassurance for people considering this treatment.

**Summary**

- Significant outcomes
  ○ People who received ECT had no statistically significant increase in risk for dementia.
  ○ There was a slight reduction in mortality after a mean of 24.5 years follow‐up.
- Limitations
  ○ Since the electronic health records in Wales are reliable from late 1990s, they do not cover earlier hospitalisations for affective disorders, therefore the actual age at onset of these disorders is likely to be earlier than reported in this work.
  ○ ECT is usually reserved for people with severe or treatment‐resistant depression. We didn't have information on detailed illness characteristics that indicate a more severe presentation of illness and could only use the best proxy information that we had for severe illness, namely the number of hospitalisations with a primary diagnosis of affective disorders and the age of the people at their first hospitalisations. This could result in under‐correction for illness severity and lead to estimates of higher risk than if a better‐matched group could be constructed.
  ○ Finally, we did not analyse other psychiatric disorders, such a schizophrenia, as the numbers were much smaller to allow for meaningful statistical analysis.

## 1 Introduction

Electroconvulsive therapy (ECT) is the most effective treatment for severe, psychotic or treatment‐resistant depression [1, 2, 3, 4]. Subjective memory problems are a common side effect [5], but cognitive test scores usually improve within a few weeks after the end of the treatment [6]. However, longer‐term cognitive consequences are also reported and are less well studied [1, 7]. Many ECT users are worried about their long‐term cognitive performance after experiencing memory problems [8]. There is a long‐standing debate about whether ECT causes brain damage, which has been largely disproved but continues to worry ECT recipients [9]. If ECT causes brain damage, one consequence could be earlier onset of dementia.

The risk of dementia in people who have received ECT has been assessed in three large, population‐based studies: two from Denmark and one from Taiwan. Osler et al. [10] used the Danish hospital registers and reported on 5901 patients who had ECT for affective disorders. They were matched in a case‐control design with 162,114 controls with similar psychiatric conditions, who did not receive ECT, and were followed up for a median of 4.9 years. The rate of dementia during follow‐up was 3.6% in the ECT group and 3.1% in controls, which, after correcting for relevant confounders, resulted in a non‐significant hazard ratio of 0.98. Hjerrild et al. [11] also used the Danish registers but focused on the longer‐term risk and analysed the histories of patients who had received ECT for affective disorders at Aarhus University Hospital between 1982 and 2000. They identified 1089 ECT‐treated patients, matched them with 3011 patients hospitalised with similar conditions, and followed them up for a median of 15 years (maximum of 34 years). The cumulative incidence of dementia was 13.4% versus 10.5% in the ECT and non‐ECT groups respectively. The adjusted hazard ratio for developing dementia was numerically higher in the ECT group but the difference was not significant: HR = 1.37 (0.96–1.92). The third study was based on Taiwan's National Health Insurance Research Database [12]. The authors identified 994 patients treated with ECT and matched them to 2982 controls not treated with ECT. This cohort is different from the ones from Denmark, as it included a large proportion of patients suffering with schizophrenia (46.7%), reflecting the different indications for ECT in Asian countries, whereas depression is by far the most common indication for ECT in Europe [13]. After a 10‐year follow‐up, 4.5% of ECT cases and 5.0% of the controls developed dementia. After controlling for confounders, this translated to a reduced hazard ratio of incident dementia in the ECT group of 0.63 (0.44–1.9) but the difference was not significant. Overall, these studies found no evidence that ECT increases risk for dementia, but the 95% confidence intervals were large, the trends were in different directions, and it was clear that adequate control for confounding between ECT‐exposed versus ECT‐unexposed groups is a critical and challenging step.

Dementia risk is increased in people with psychiatric disorders and the risk is higher for more severe disorders, such as schizophrenia and bipolar disorder, compared to depression or anxiety disorders, as reported previously [14, 15, 16], and by our group on the population of Wales [17]. ECT is usually offered to the most severely ill patients and therefore it is conceivable that such patients have an even higher risk for dementia, making the accurate control for confounding in such studies problematic and only partial.

## 2 Aims of the Study

The different populations examined in the previous studies, the different designs and follow‐up periods in these studies, and the different trends require further studies to examine the risk for dementia after ECT. We wanted to use the UK NHS clinical datasets of the entire population of Wales in order to assess whether a history of ECT is associated with an increased risk for developing dementia.

## 3 Materials and Methods

We conducted an observational cohort study in Wales, using the UK NHS administrative and clinical datasets for primary and secondary care records stored in the Secure Anonymised Information Linkage (SAIL) databank https://saildatabank.com/. SAIL contains anonymised individual‐level health records for about 4.4 million people, covering about 85% of the Welsh population for primary care (general practitioners') data and 100% for hospital data (https://saildatabank.com/the‐value‐of‐sail‐databank‐for‐clinical‐trials/). Our study used data under the approved SAIL application #0998.

The SAIL databank [18] was originally a repository of health data but has expanded to include administrative data. Individuals do not enrol in the study; thus, SAIL provides a representative population‐level sample. Ethical approval to perform the current study was provided by the Information Governance Review Panel (IGRP) of SAIL. All data contained in SAIL has the permission for use from the relevant Caldicott Guardian or Data Protection Officer. All analyses were carried out in accordance with the relevant guidelines and regulations.

Data were taken from the Welsh Longitudinal General Practitioner Dataset (WLGP), Patient Episode Dataset for Wales (PEDW), Outpatient Database for Wales (OPDW) and Welsh Demographic Services Dataset (WDSD) data tables. Information on demographics, including place of residence (Wales or other), sex, date of birth and date of death were taken from WDSD and WLGP. Information on treatment and diagnoses was taken from WLGP and PEDW. Measures of deprivation were taken from the WDSD dataset, using the Welsh Index of Multiple Deprivation (WIMD) 2014 statistics (https://www.gov.wales/sites/default/files/statistics‐and‐research/2019‐04/welsh‐index‐of‐multiple‐deprivation‐2014‐revised.pdf). The WIMD is designed to identify small areas where there are the highest concentrations of several different types of deprivation. It is made up of eight separate domains, each compiled from a range of different indicators: income, employment, health, education, access to services, community safety, physical environment and housing. Individuals' most recent deprivation score was used to assign a decile value. Individuals without information on place of residence, or who were resident outside of Wales, were removed from the dataset.

Individuals were selected if they were alive and aged between 35 and 65 on 1.1.1995. This age range was chosen to ensure that they had a reasonable chance of developing dementia before the end of the study period when the youngest person would be 65 years of age, and to reduce the number of people who might have developed dementia before reliable records became available in 1995. Individuals with a recorded diagnosis of dementia before 1995 were excluded. The total number of people remaining after these initial filtering steps was 1,412,926.

Dementia cases were defined as individuals who had a first diagnosis of dementia during the study period in either the hospital datasets (PEDW, OPDW), using ICD‐10 codes, or from the primary care (WLGP) dataset, using UK National Health Service (NHS) diagnostic codes (CV2 read codes), as people with dementia are not necessarily admitted to hospital. The following ICD‐10 codes from the hospital datasets were used for identifying dementia: Alzheimer's disease (F00, G30), vascular dementia (F01) and unspecified dementia (F03). We did not include the category G31 (other degenerative diseases of the nervous system) that includes frontotemporal dementia (G31.0) and Lewy body dementia (G31.83). The following CV2 read codes (used for primary care, WLGP) were used: Alzheimer's disease (Eu00, F110), vascular dementia (Eu01) and unspecified dementia (Eu02z).

Affective disorders are the most common indication for receiving ECT in the UK [19] and we limited the analysis to such patients. As ECT is normally given to the more severely ill patients, in order to compare as closely as possible those who received ECT with persons who didn't, we only included people who had been hospitalised with such diagnoses during the study period. Affective disorder diagnoses were extracted from hospital records (PEDW dataset). We included the following ICD10 codes for affective disorders: mania, bipolar disorder, single or recurrent depressive episode and persistent mood affective disorders (F30‐F34). We did not exclude patients who, on different occasions, had been diagnosed with other psychiatric diagnoses. The maximum number of hospitalisations was truncated to 15, as there were a small number of people with very high number of hospitalisations.

Information on ECT treatment was extracted from hospital records (PEDW, OPDW datasets) using the OPSC‐4 (NHS digital) codes A83.8 and A83.9 (https://classbrowser.nhs.uk/#/), which correspond to electroconvulsive therapy in hospital records.

We used an established comorbidity measure (Charlson comorbidity index (CCI)) calculated as the number of comorbid disorders per individual, which is frequently used for risk adjustment by healthcare researchers [20]. ICD10 codes were used to generate CCI, which include diagnoses of liver disease, tumours, diabetes, rheumatic disease, congestive heart failure, pulmonary disease, cerebrovascular disease, peptic ulcer disease, hemiplegia and paraplegia, renal disease and myocardial infarction at any point.

In our primary analysis to estimate the hazard ratio for the association between exposure to ECT at any point during the study period and incident dementia, we used Cox proportional hazards models. Individuals were followed from cohort entry in 1995 until incident dementia diagnosis, with censoring occurring on death, the end of the study period, or emigration from Wales. These models adjusted for potential confounders including psychiatric diagnosis, sex, social deprivation index, CCI, age at first affective disorder hospitalisation, the number of hospitalisations for affective disorders and history of severe alcohol abuse, defined as hospitalisation for mental and behavioural disorders due to use of alcohol (F10). This analytic approach estimates the cause‐specific hazard ratio, which is recommended for studying etiologic questions [21]. Given that death may be a competing risk in our primary analytic model, we assessed how robust our analysis was in accounting for the competing risk of death (people who die early have a reduced chance of developing dementia). We conducted a secondary analysis estimating the hazard ratio for the association between ECT and dementia using the Fine‐Gray regression method [22] which models the impact of covariates on the cumulative incidence of an event of interest in the presence of competing risks, adjusting for the same confounders as our primary analytic model. Risk of death (all‐cause mortality) was assessed also with Cox proportional hazards model.

## 4 Results

The number of people who were hospitalised with a primary diagnosis of affective disorders during the study period was 110,774. The majority of those (*N* = 105,362) had diagnoses of single or recurrent depressive episode at their first recorded hospitalisation (F32 and F33), whereas smaller numbers had mania/bipolar disorder (F30‐F31), *N* = 5002 or persistent mood affective disorders (F34), *N* = 410. The mean duration of follow‐up was 24.5 years (SD = 6.3). Of the 110,774 people, 1033 received ECT during the study period. Of those, 23 had received a diagnosis of dementia at least 12 months prior to the date given for the ECT course, and they were included in the non‐ECT group, leaving 1010 ECT‐treated patients. (In order to be conservative in our analysis we allowed a 12‐month window, avoiding potential inaccuracies in the recorded dates of entries of these events into the databases). The mean duration of follow‐up after the first course of ECT was 12.0 years (SD = 8.3).

**FIGURE 1:**
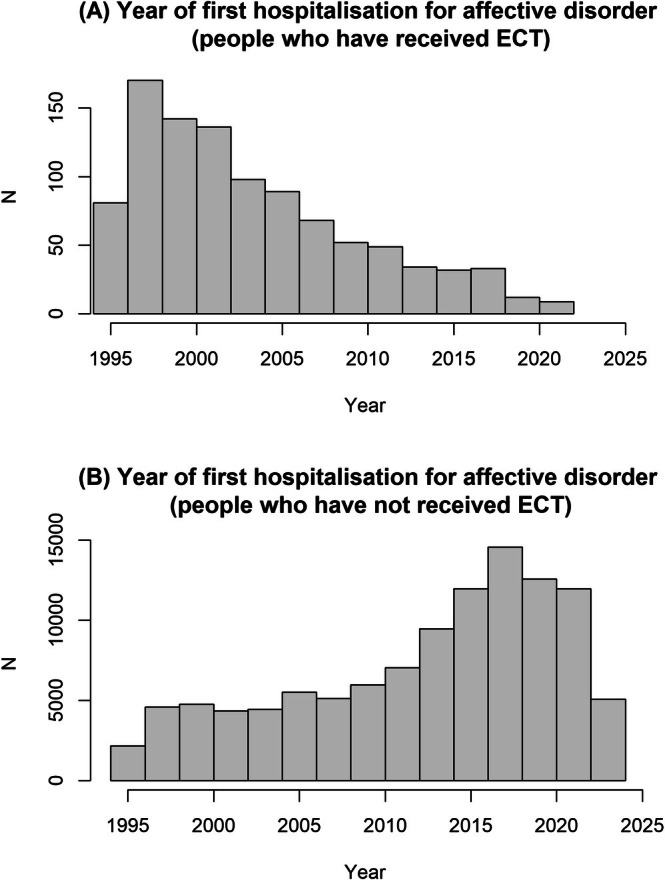
Number of people diagnosed with dementia during the follow‐up interval.

The characteristics of the patients in the two groups of this study (those with and without ECT history) are presented in Table 1. Although both groups were hospitalised for affective disorders, there remained notable differences: several variables have standardised mean differences of greater than 0.1, which is often considered a sign of important covariate imbalance [23]. Thus, patients who received ECT were older in 1995, had more hospitalisations and these occurred at a much earlier age (Table 1, Figure 1), suggesting that they had more severe courses of illness.

**TABLE 1:** Characteristics of people in the study, divided by history of ECT.

Total *N* hospitalised with affective disorders: *N* = 110,774	Had ECT before dementia	Did not have ECT before dementia	Standardised difference
*N* individuals	1010	109,764	
*N* females	627 (62.1%)	65,817 (60.0%)	0.043
Mean age in 1995 (SD)	50.0 (8.7)	48.3 (8.7)	0.192
Mean age at first ECT (SD)	60.2 (10.4)	n/a	n/a
Charlson comorbidity index (CCI) (SD)	1.39 (1.7)	1.49 (1.7)	0.060
Welsh index of multiple deprivation (WIMD)	2.92 (1.3)	2.82 (1.4)	0.070
Mean *N* of hospitalisations with affective disorder (SD)	6.79 (5.0)	2.37 (2.6)	1.099
Mean age at first hospitalisations with affective disorder (SD)*	58.8 (10.4)	66.0 (11.1)	0.676
Hospitalisation for alcohol abuse**	134 (13.3%)	13,852 (12.6)	0.019
*N* with dementia	156 (15.4%)	14,360 (13.1%)	0.068
Mean age at dementia diagnosis (SD)	72.1 (9.4)	74.5 (9.4)	0.248
*N* dead	576 (57.0%)	50,307 (45.8%)	0.225
Mean age at death (SD)	72.8 (9.5)	73.7 (9.8)	0.093

* The true age at onset of affective disorder is likely to be much earlier, but we only used data on hospitalisations with affective disorders after 1995.

** History of hospitalisation for mental and behavioural disorders due to use of alcohol (F10).

Of the 110,774 people hospitalised with affective disorders, 14,516 were diagnosed with dementia by the end of the study (13.1%). Of those, 156 had received ECT, giving a rate of dementia of 15.4% in the ECT group (Table 1).

**FIGURE 2:**
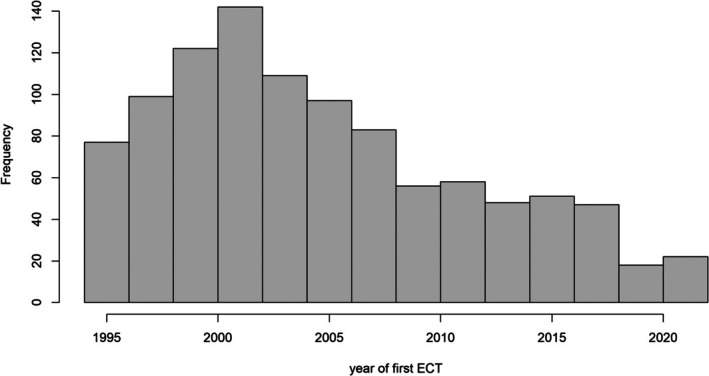
Number of first time ECT courses during the study period.

Figure 2 shows the number of first time ECT courses given during the study period. There was a steep reduction in incident ECT use over the 30‐year period.

We performed Cox proportional hazards model for the risk of dementia (Table 2). We failed to find a statistically significant association between ECT exposure and dementia after the inclusion of the confounders (HR = 0.888; 95% CI: 0.757–1.044, *p* = 0.15), with the survival curves for the two groups practically overlapping (Figure S1). All included covariates had significant associations with the risk of incident dementia. The HRs were increased for men, people with higher deprivation, more medical comorbidities, an earlier age at psychiatric diagnosis, a higher number of hospitalisations for affective disorders, and a history of hospitalisations for mental and behavioural disorders due to use of alcohol (F10).

**TABLE 2:** Cox proportional hazards model results: Hazard ratios (HRs) for the risk of dementia in people with and without ECT accounting for sex, Welsh index of multiple deprivation, Charlson comorbidity index, age at first hospitalisation with a diagnosis of affective disorders, the number of such hospitalisations, and history of hospitalisations for alcohol abuse.

	HR	95% CI	*p*
ECT	0.888	(0.757–1.044)	0.15
Covariates			
Female sex	0.927	(0.896–0.959)	1.3 × 10^−5^
Welsh index of multiple deprivation*	0.969	(0.958–0.980)	1.2 × 10^−7^
Charlson comorbidity index	1.036	(1.027–1.046)	8.8 × 10^−14^
Affective disorder hospitalisation age	0.983	(0.981–0.985)	1.6 × 10^−72^
Number of hospitalisations for affective disorders	1.041	(1.036–1.047)	2.7 × 10^−45^
Alcohol abuse**	1.794	(1.707–1.885)	3.6 × 10^−118^

* Larger index corresponds to less deprived area.

** History of hospitalisation for mental and behavioural disorders due to use of alcohol (F10).

We then used the Fine‐Gray regression method [22] as our secondary (sensitivity) analysis to estimate the HR for the association between ECT and dementia, with death as a competing risk (Methods). The HR for dementia remained practically unchanged and non‐significant (HR = 1.104, 95% CI: 0.945–1.289, *p* = 0.21, Table S1).

The study by Osler et al. from Denmark [10] reported that with higher age ECT could even be associated with a reduced risk of dementia. We therefore analysed our results by dividing the cohort into two groups: aged 50 years and less and 51 years and more in 1995 (Table S2). We replicate the results by Osler et al. [10] by observing a significant reduction in the HR for the older population (HR = 0.741, 95% CI = 0.60–0.91, *p* = 0.004). The younger group, however, had an increased HR = 1.399 (95% CI = 1.08–1.82), which reached nominal levels of statistical significance (*p* = 0.012, Table S3A,B). This observation replicates a similar, but not significant trend in younger people reported by Osler et al. [10].

In crude analyses, the risk of death among the ECT group was higher: 57.0% of those who had ECT had died by 2024, compared to 45.8% among those who had not had ECT. We performed the same Cox proportional hazards model as above, this time using death as the outcome. ECT was no longer associated with an increased risk of death, whereas most other covariates had highly statistically significant associations, in the same directions as for dementia (Table 3). In fact, the HR for ECT was significantly reduced at 0.81, *p* = 7.4 × 10^−7^.

**TABLE 3:** Cox proportional hazards model results for association of ECT with risk of death in people with and without ECT accounting for sex, Welsh index of multiple deprivation, Charlson comorbidity index, age at hospitalisation with a diagnosis of affective disorders, the number of such hospitalisations and history of hospitalisation for alcohol abuse.

	HR	95% CI	*p*
ECT	0.810	(0.745–0.880)	7.4 × 10^−7^
Covariates			
Female sex	0.761	(0.747–0.775)	4.7 × 10^−198^
Welsh index of multiple deprivation*	0.925	(0.919–0.930)	9.4 × 10^−132^
Charlson comorbidity index	1.085	(1.080–1.090)	1.9 × 10^−236^
Affective disorder hospitalisation age	0.940	(0.939–0.941)	< 2.2 × 10^−308^
Number of hospitalisations for affective disorders	0.998	(0.994–1.001)	0.13
Alcohol abuse**	1.585	(1.546–1.625)	3.9 × 10^−290^

* Lower index corresponds to more deprived area.

** History of hospitalisation for mental and behavioural disorders due to use of alcohol (F10).

## 5 Discussion

We studied a population‐wide cohort of people for the potential effect of ECT on the development of dementia. We selected patients aged between 35 and 65 on 1.1.1995, around the time when reliable electronic hospital data became available in Wales. After that year we have nearly complete hospital data, including information on ECT. The data conforms with independently collected statistics on the use of ECT in the UK in 2021 [19] which reported a similar proportion of females treated with ECT (67% vs. 62%), and a similar age of people given ECT (62.1 vs. 60.4 years). The distribution of ECT courses over the years (Figure 2) mirrors independent reports that found more than a five‐fold reduction in the use of ECT in the UK over the last 25 years; from around 11,000 people in 1999 to below 2000 in 2021 [19, 24].

People who have a history of psychiatric disorders have a highly increased rate of dementia, and this risk increases in patients with more severe psychiatric disorders such as schizophrenia or bipolar disorder, compared to less severe conditions, such as anxiety [17]. Therefore, it is logical to assume that patients with severe psychiatric disorders (i.e., who are more likely to be treated with ECT) would have an even higher risk for developing dementia. Indeed, ECT patients had a 15.4% rate of developing dementia during the 24.8 years follow‐up. For comparison, only 5.5% (*N* = 78,305) of the ~1.4 million people of this age range had developed dementia during this period. In order to construct a more homogenous sample, we decided to analyse only patients hospitalised for psychiatric disorders. As over 90% of the indications for ECT in the UK are for affective disorders [19], we also restricted the analysis to the 110,774 people hospitalised with these diagnoses. The rate of dementia among such people was highly increased, at 13.1%. However, this is still likely to be a much less severely ill population compared to those who received ECT, as ECT tends to be given to some of the most severely ill patients in the UK, with about a third of them having psychotic depression and a high rate having severe self‐neglect, poor food intake or suicide risk [19]. Indeed, ECT patients in the current study had an earlier age at first hospitalisation and had more admissions to hospital with psychiatric disorders (Table 1 and Figure 1).

We therefore performed Cox proportional hazards analysis, using as covariates age and sex, as well as further demographic and medical outcomes that can influence the risk of dementia: medical comorbidities (Charlson comorbidity index), social deprivation (Welsh index of multiple deprivation), age at first hospitalisation, the number of hospitalisations with a primary diagnosis of affective disorders and hospitalisations for mental and behavioural disorders due to alcohol abuse. The correction for all these factors resulted in no statistically significant association with developing dementia (Table 2 and Figure S1). The association with dementia was even reduced in the more elderly population, replicating an observation made in a previous study [10]. The increased HR among the younger group reflects, in our opinion, the insufficient controlling for the severity of their illness, thus ignoring the fact that ECT is given only to the most severely ill hospitalised patients. Having ECT earlier in life is an indicator of a more severe and refractory depression course, which might increase the risk for dementia because of the repeated or prolonged depressive episodes. This reflected the trend reported in the study by Osler et al. [10].

Our results are overall similar in terms of dementia risk (i.e., no association) to the previous reports discussed in the Introduction and support the overall conclusion of a lack of statistically significant association with dementia after ECT. This is important information for people considering ECT because this treatment causes memory problems in a substantial proportion of patients, and they can be worried that this can lead to a cognitive decline and even dementia. People treated with ECT can have persistent memory problems that can be distressing and impair their functioning, but it does not appear that ECT increases the risk of a major neurocognitive disorder. This conclusion is supported by a large meta‐analysis of performance on cognitive tests, which are even improved compared to pre‐ECT baseline if tests are done more than 15 days after the last ECT [6], and by the lack of cumulative cognitive deficits after repeated courses of ECT [25]. Patients and carers need to be aware of the increased rate of dementia among people with severe psychiatric disorders and that this risk does not appear to be increased by treatment with ECT.

Our study found no increased risk for mortality once we corrected for the same factors as for the analysis for dementia risk (Table 3). In fact, the HR for ECT‐treated patients was even reduced at 0.81 (95% CI = 0.745–0.880, *p* = 7.4 × 10^−7^). This reduced risk is very similar to the long‐term risk reported in recent studies from Denmark, Canada, and the US [10, 26, 27] which found no increase or even reduced long‐term risk of death after ECT. This reduction in risk is likely due to several factors. For example, there is potential inherent bias in the analysis that results from clinicians not giving ECT to patients with severe physical problems (e.g., previous myocardial infarction) [10], although in the current study the two groups had a similar comorbidity index (CCI, Table 1). There is also a reduced risk of suicide following ECT [26]. It is also possible that the positive effect of ECT on depression reduces mortality, as depression itself is strongly associated with increased mortality [27]. Whatever the reasons, the reduced risk of death is another reassuring finding for ECT patients.

## 6 Conclusions

This register‐based study on the whole population of Wales failed to detect an increased association with dementia in people treated with ECT. We find a reduction in the longer‐term risk of death, with a hazard ratio of 0.81 (95% CI: 0.745–880), similar to previous studies. Our findings should provide a further reassurance on the long‐term safety of this treatment.

## Author Contributions

George Kirov participated in the conception and design of the work, wrote the first draft of the manuscript, critically reviewed the manuscript. Emily Simmonds analysed the data. Tyler Kaster contributed to interpretation and statistical analysis of data and critically reviewed the manuscript. Valentina Escott‐Price participated in conception and design of the work, analysed and interpreted the data, drafted and reviewed the manuscript. All authors approved the final version.

## Disclosure

Transparency Declaration: We affirm that the manuscript is an honest, accurate, and transparent account of the study being reported; no important aspects of the study have been omitted.

## Conflicts of Interest

The authors declare no conflicts of interest.

## Peer Review

The peer review history for this article is available at https://www.webofscience.com/api/gateway/wos/peer‐review/10.1111/acps.70005.

## Supporting information


**Figure S1.** Probability of not receiving a dementia diagnosis in ECT‐treated and patients in those who did not receive ECT, using Cox proportional hazards models. The two survival lines almost overlap.
**Table S1.** Fine and Gray model analysis for the risk of dementia in which death is a competing risk. Sub‐distribution hazard ratios (HRs) for the risk of dementia in people with and without history of ECT accounting for sex, Welsh Index of Multiple Deprivation, Charlson Comorbidity Index, age of first hospitalisation with affective disorders, the number of such hospitalisations and history of hospitalisations for alcohol abuse.
**Table S2.** Characteristics of people, divided by history of ECT and age (below and above 50 years old on 1.1.1995).
**Table S3.** Cox proportional hazards model results in the two groups aged 50 years and less (A) and 51 year and more (B) in 1995: HRs (exp(B)) for the Hazard Ratios (HR) of dementia in people with and without ECT accounting for sex, Welsh Index of Multiple Deprivation, Charlson Comorbidity Index, age at first hospitalisation for affective disorders, the number of hospitalisation for depression and history of alcohol abuse.

## Data Availability

No datasets were generated during the current study. Raw data are available following application to the SAIL databank (https://saildatabank.com/). The analytic code supporting the findings is available within the SAIL virtual platform but can be shared upon request to the authors. Note that these codes are specific to the data within the virtual platform. There are no generated data to make available.
