# Supplementary material for: Risk of Dementia After Electroconvulsive Therapy: A Cohort Study on the Population of Wales
Source: Acta Psychiatr Scand. 2025 Jun 19;152(4):270–7. doi: 10.1111/acps.70005 (PMC12404881; doi:10.1111/acps.70005)
Supplement: Supplementary file 1 — Figure S1. Probability of not receiving a dementia diagnosis in ECT‐treated and patients in those who did not receive ECT, using Cox proportional hazards models. The two survival lines almost overlap. Table S1. Fine and Gray model analysis for the risk of dementia in which death is a competing risk. Sub‐distribution hazard ratios (HRs) for the risk of dementia in people with and without history of ECT accounting for sex, Welsh Index of Multiple Deprivation, Charlson Comorbidity Index, age of first hospitalisation with affective disorders, the number of such hospitalisations and history of hospitalisations for alcohol abuse. Table S2. Characteristics of people, divided by history of ECT and age (below and above 50 years old on 1.1.1995). Table S3. Cox proportional hazards model results in the two groups aged 50 years and less (A) and 51 year and more (B) in 1995: HRs (exp(B)) for the Hazard Ratios (HR) of dementia in people with and without ECT accounting for sex, Welsh Index of Multiple Deprivation, Charlson Comorbidity Index, age at first hospitalisation for affective disorders, the number of hospitalisation for depression and history of alcohol abuse. [file ACPS-152-270-s001.docx]

**Supplementary Material**

**Supplementary Figure 1.** Probability of not receiving a dementia diagnosis in ECT-treated and patients in those who did not receive ECT, using Cox proportional hazards models. The two survival lines almost overlap.


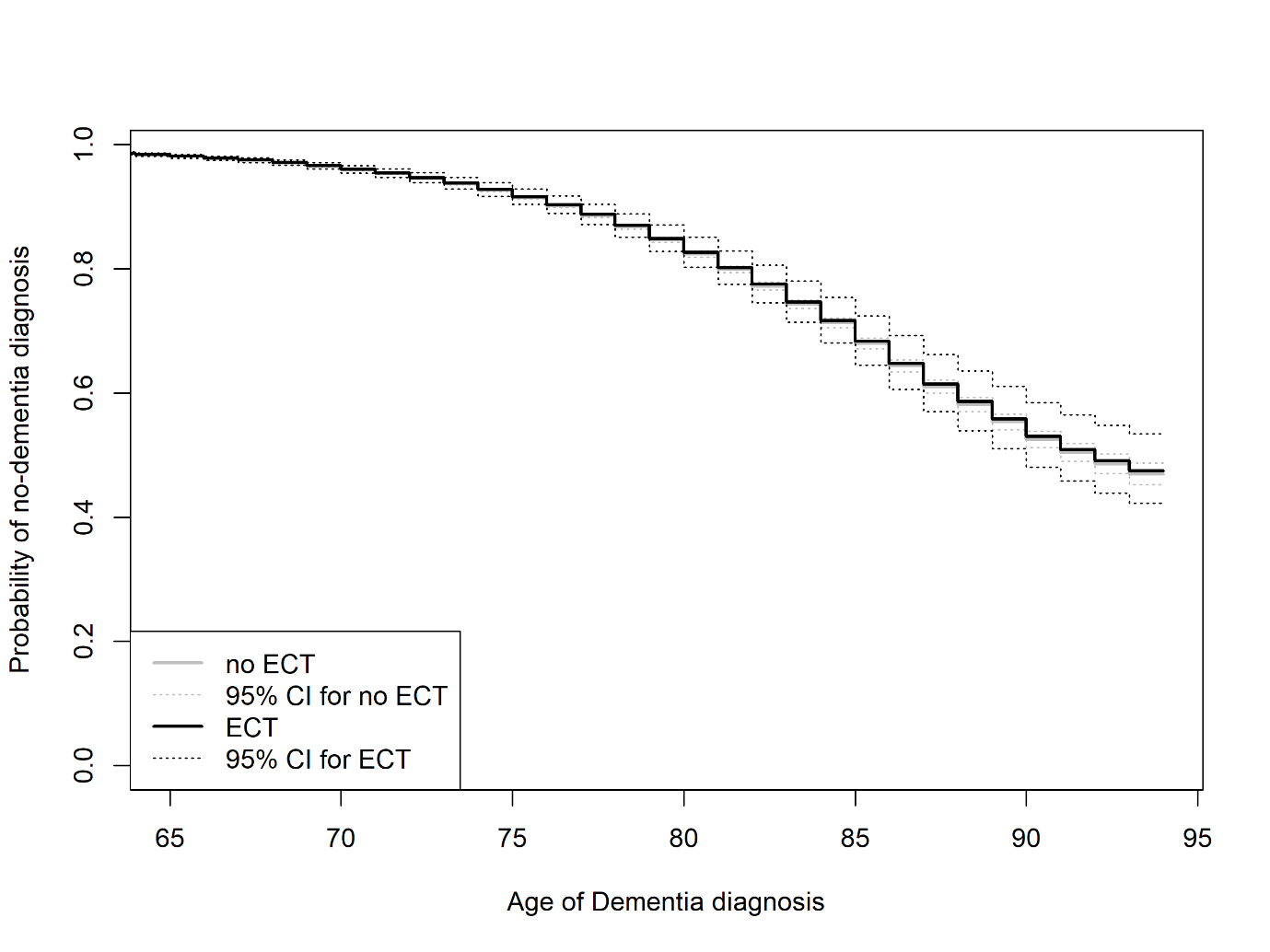


**Supplementary Table 1.** Fine & Gray model analysis for the risk of dementia in which death is a competing risk. Sub-distribution hazard ratios (HRs) for the risk of dementia in people with and without history of ECT accounting for sex, Welsh Index of Multiple Deprivation, Charlson Comorbidity Index, age of first hospitalisation with affective disorders, the number of such hospitalisations and history of hospitalisations for alcohol abuse.

|  | HR | 95% CI | p-value |
| --- | --- | --- | --- |
| ECT | 1.104 | [0.945-1.289] | 0.21 |
| Covariates: |  |  |  |
| Female sex | 1.030 | [0.998-1.064] | 0.07 |
| Welsh Index of Multiple Deprivation* | 0.998 | [0.987-1.009] | 0.77 |
| Charlson Comorbidity Index | 1.018 | [1.009-1.027] | 0.00012 |
| Affective disorder hospitalisation age | 1.028 | [1.027-1.030] | 0 |
| Number of hospitalisations for affective disorders | 1.049 | [1.044-1.054] | 1.2x10^-86^ |
| Alcohol abuse** | 1.498 | [1.428-1.571] | 6.4x10^-62^ |

* Larger index corresponds to less deprived area

** History of hospitalisation for mental and behavioural disorders due to use of alcohol (F10)

**Supplementary Table 2.** Characteristics of people, divided by history of ECT and age (below and above 50 years old on 1.1.1995)

| Total N hospitalised with depression: N=110,774 | had ECT  age ≤50 | did not  have ECT  age ≤50 | had ECT  age >50 | did not  have ECT  age >50 |
| --- | --- | --- | --- | --- |
| N individuals | 538 | 66,364 | 472 | 43,400 |
| N Females | 320 (59.5%) | 39,124 (59.0%) | 307 (65%) | 26,693 (61.5%) |
| Mean age in 1995 [SD] | 43.0 [4.6] | 42.3 [4.6] | 57.9 [4.3] | 57.5 [4.3] |
| Mean age at first ECT [SD] | 53.8 [8.4] | n/a | 67.6 [7.2] | n/a |
| Charlson comorbidity index (CCI) [SD] | 1.31 [1.7] | 1.34 [1.6] | 1.48 [1.72] | 1.72 [1.76] |
| Welsh Index of Multiple Deprivation (WIMD) | 2.97 [1.3] | 2.75 [1.4] | 2.86 [1.31] | 2.94 [1.40] |
| Mean N of hospitalisations  with affective disorder [SD] | 7.00 [5.1] | 2.45 [2.8] | 6.56 [5.0] | 2.25 [2.4] |
| Mean age at first hospitalisations with affective disorder [SD] | 52.0 [8.1] | 60.4 [8.8] | 66.4 [6.8] | 74.6 [8.42] |
| Hospitalisation for alcohol abuse | 90 (16.7%) | 10,344 (15.6%) | 44 (9.3%) | 3,508 (8.3%) |
| N with dementia | 60 (11.2%) | 3899 (5.9%) | 96 (20.3%) | 10,461 (24.1%) |
| Mean age at dementia diagnosis [SD] | 64.6 [9.2] | 64.6 [8.4] | 76.8 [5.9] | 78.2 [6.6] |
| N dead | 210 (39.0%) | 20,511 (30.9%) | 366 (77.5%) | 29,796 (68.6%) |
| Mean age at death [SD] | 65.0 [8.0] | 65.6 [7.3] | 77.2 [7.0] | 79.2 [7.1] |

**Supplementary Table 3.** Cox proportional hazards model results in the two groups aged 50 years and less (A) and 51 year and more (B) in 1995: HRs (exp(B)) for the Hazard Ratios (HR) of dementia in people with and without ECT accounting for sex, Welsh Index of Multiple Deprivation, Charlson Comorbidity Index, age at first hospitalization for affective disorders, the number of hospitalizations for depression and history of alcohol abuse.

A

| Age 50 years and less in 1995 | HR | 95% CI | p-value |
| --- | --- | --- | --- |
| ECT | 1.399 | [1.08-1.82] | 0.012 |
| Sex | 0.807 | [0.76-0.86] | 3.7x10^-11^ |
| Welsh Index of Multiple Deprivation (WIMD) | 0.969 | [0.95-0.99] | 6.6x10^-3^ |
| Charlson comorbidity index (CCI) | 1.136 | [1.12-1.16] | 6.9x10^-46^ |
| Age at first hospitalisations with affective disorder | 0.991 | [0.987-0.994] | 3.3x10^-6^ |
| Number of hospitalisations for depression | 1.051 | [1.04-1.06] | 3.4x10^-27^ |
| Hospitalisation for alcohol abuse | 2.274 | [2.11-2.45] | 1.7x10^-105^ |

B

| Age 51 years and more in 1995 | HR | 95% CI | p-value |
| --- | --- | --- | --- |
| ECT | 0.741 | [0.60-0.91] | 0.004 |
| Sex | 0.982 | [0.94-1.02] | 0.372 |
| Welsh Index of Multiple Deprivation (WIMD) | 0.972 | [0.96-0.99] | 4.7x10^-5^ |
| Charlson comorbidity index (CCI) | 1.001 | [0.99-1.01] | 0.863 |
| Age at first hospitalisations with affective disorder | 0.981 | [0.978-0.983] | 3.9x10^-54^ |
| Number of hospitalisations for depression | 1.031 | [1.02-1.04] | 7.8x10^-17^ |
| Hospitalisation for alcohol abuse | 1.430 | [1.33-1.53] | 1.1x10^-23^ |
